# Metabolic Syndrome in Type 2 Diabetes Mellitus Patients: Prevalence, Risk Factors, and Associated Microvascular Complications

**DOI:** 10.7759/cureus.39076

**Published:** 2023-05-16

**Authors:** Shoaib Asghar, Sohaib Asghar, Salman Shahid, Mishal Fatima, Syed Muhammad Hassan Bukhari, Simra Nadeem Siddiqui

**Affiliations:** 1 Internal Medicine, Shaikh Zayed Medical College and Hospital, Rahim Yar Khan, PAK; 2 Gastroenterology, Glan Clwyd Hospital, Betsi Cadwaladr University Health Board, Rhyl, GBR; 3 Internal Medicine, Bedfordshire Hospitals NHS Foundation Trust, Bedford, GBR; 4 Internal Medicine, King’s College Hospital NHS Foundation Trust, London, GBR; 5 Emergency Medicine, Glan Clwyd Hospital, Betsi Cadwaladr University Health Board, Rhyl, GBR

**Keywords:** high-density lipoprotein-cholesterol (hdl-c), body mass index (bmi), diabetic retinopathy, diabetic neuropathy, diabetic nephropathy, type 2 diabetes mellitus (t2dm), hypertriglyceridemia (tg), hypertension, abdominal obesity, metabolic syndrome (metsy)

## Abstract

Background

The chronic macro and microvascular complications of diabetes mellitus pose serious health challenges. Metabolic syndrome (MetSy) is characterized by central obesity, glucose intolerance, hyperinsulinemia, low high-density lipoproteins (HDLs), high triglycerides (TGs), and hypertension. MetSy precedes or accompanies diabetes, and it has been linked to an increased risk of cardiovascular disease and premature death. This study aimed to estimate prevalence, identify risk factors, and evaluate associated microvascular complications among MetSy patients with type 2 diabetes mellitus (T2DM).

Methodology

Over the period of March 20, 2022, to March 31, 2023, a prospective cohort study was conducted at the Outdoor Clinic and Medicine Department of Sheikh Zayed Hospital, Rahim Yar Khan. Based on the International Diabetes Federation MetSy criteria, a total of 160 patients fulfilling the inclusion criteria were selected.

A special proforma was used to obtain sociodemographic, clinical, and laboratory variables of MetSy in diabetic participants. Blood pressure and anthropometric measurements such as waist circumference (WC) and body mass index (BMI) were measured. Fasting venous blood was collected to analyze biochemical variables such as fasting blood sugar (FBS), TG, and high-density lipoprotein-cholesterol (HDL-C). The microvascular complications of T2DM were established using fundus ophthalmoscopy and neurological and kidney function assessments with the help of laboratory tests. These variables were matched between MetSy and no MetSy groups along with the presence or absence of diabetes microvascular complications. This information was analyzed based on these assessments and patient interviews.

Results

Of the 160 T2DM patients, the mean age was 52 years with a predominance of females (51.8%) in the 50-59-year age group (56.8%). The average BMI for females was 29.38 ± 0.54 kg/m², and 32 (20%) had obesity. Females exhibited a large WC of 93.52 ± 1.58 cm, and 48 of 83 females had reported diabetes microvascular complications. A significant p-value was observed for hypertension, high TG, low HDL-C, large WC, obesity, BMI, age, and female gender on comparing diabetics with metabolic syndrome (MetSy+) and those without metabolic syndrome (MetSy-).

The prevalence of microvascular complications in T2DM patients with MetSy+ was 52.5% compared with 47.5% in MetSy-. The prevalence of diabetic retinopathy was 24.9% (95% confidence interval (CI) = 20.3%-29.6%), nephropathy was 16.8% (95% CI = 12.8%-20.7%), and neuropathy was 10.8% (95% CI = 7.4%-13.3%).

Conclusions

The prevalence of MetSy observed among T2DM patients was 65%, with married obese females in the 50-59-year age group being more likely to be affected than males. Hypertension, poor glycemic control, high TG, low HDL-C, and greater anthropometric waist measurements and BMI were additional risk factors that tended to increase the MetSy burden in T2DM. Diabetic retinopathy, nephropathy, and neuropathy were the most prevalent microvascular complications of diabetes, and immediate attention is needed to stop their detrimental effects. Longer uncontrolled diabetes, increasing age, and hypertension were independent predictors of microvascular complications.

To further reduce the risks of complications that threaten healthy aging and prognosis for these patients, MetSy screening, health education, and better diabetic management are crucial.

## Introduction

As one of the most common metabolic diseases, diabetes mellitus poses a serious public health concern globally because of its chronic microvascular and macrovascular complications. The International Diabetes Federation Obesity Task Force (IOTF) reports that 1.7 billion people globally are at risk of weight-related, non-communicable diseases such as type 2 diabetes mellitus (T2DM) [1]. Metabolic syndrome (MetSy) is a constellation of central obesity, glucose intolerance, hyperinsulinemia, low high-density lipoproteins (HDLs), high triglycerides, and hypertension [2]. In 70%-80% of diabetics, MetSy precedes or accompanies diabetes [3], and it has been linked to a threefold increased risk of cardiovascular disease (CVD) and premature death [4].

Besides resulting in macrovascular complications of T2DM, the pathological changes in microvasculature are a result of uncontrolled high blood sugar and genetic susceptibility, leading to microvascular complications in vital organs such as the kidneys (nephropathy), eyes (retinopathy), and nervous system (neuropathy) [5,6]. In the diabetes population, diabetic nephropathy is the leading cause of chronic kidney disease, diabetic neuropathy leads to diabetic foot ulcers and amputations, and diabetic retinopathy results in blindness [7,8]. Patients with T2DM suffer severe morbidity and mortality as a result of the complications associated with the disease.

Several factors determine the prevalence of MetSy, including its definition, age, gender, ethnicity, and lifestyle of the study population. The number of cases is increasing rapidly all over the world. This has been accredited to lifestyle changes with regard to sedentary routines, new eating behaviors, and physical inactivity [8]. Numerous variables have been found to be associated with microvascular complications associated with diabetes. Sociodemographic variables (age, gender, marital status, and residence), behavioral variables (physical activity, adherence to diet, obesity, and body mass index (BMI)), and clinical variables (diabetes duration, family history, glycemic control, medications, and comorbidities such as hypertension and dyslipidemia) can be identified [9-11].

According to studies, age [10], gender, marital status (single or divorced) [11], family history of diabetes [12-14], ≥5 years of diabetes duration [15-18,19-25], poor glycemic control [15], adherence to diet [16], physical inactivity [14-17], obesity [13,14], mixed medication [17], insulin therapy [19], and hypertension [4-6,8,12,25-27], were associated with the microvascular complications of diabetes.

This study aimed to assess the prevalence, identify the risk factors, and evaluate the associated microvascular complications among MetSy patients with T2DM in a public teaching hospital serving three provinces.

## Materials and methods

Operational definitions

According to the new International Diabetes Federation (IDF) [1] MetSy criteria in agreement with the American Heart Association [2], for a person to be diagnosed as having a MetSy, they must meet the below criteria.

Central obesity refers to men who have a waist circumference (WC) of at least 94 cm and women who have a WC of at least 80 cm have abdominal obesity. If the BMI is >30kg/m², central obesity can be assumed.

Plus, any two of the below four factors.

Blood pressure greater than or equal to 130/85 mmHg or treatment of previously diagnosed hypertension. Raised triglyceride level at greater than or equal to 150 mg/dL (1.7 mmol/L) or precise treatment for the lipid abnormality. Reduced high-density lipoprotein-cholesterol (HDL-C) of less than 40 mg/dL (1.03 mmol/L) for males and 50 mg/dL (1.29 mmol/L) for females or precise treatment of the lipid abnormality. Raised fasting plasma glucose (FPG) greater than or equal to 100 mg/dL (5.6 mmol/L), or previously diagnosed type 2 diabetes. oral glucose tolerance test is recommended at these values.

BMI is classified into normal weight (18.5-24.9 kg/m²), underweight (<18.5 kg/m²), overweight (25-29.9 kg/m²), and obese (>30 kg/m²) [1,2].

Physical activity refers to exercise (moderate intensity) of at least 150 minutes per week (three days) which is considered good for diabetes mellitus patients, otherwise, it is considered poor physical activity [1-3].

Glycemic control refers to fasting blood sugar (FBS) below 130 mg/dL, which is considered good control, while FBS above the indicated value is considered poor control [1-3].

Microvascular complications refer to complications of diabetes mellitus including diabetic nephropathy*, *diabetic retinopathy*, *and peripheral neuropathy [1-4] of previously or newly diagnosed diabetes.

Study design

From March 20, 2022, to March 31, 2023, a prospective cohort study was conducted at the Outdoor Clinic and Medicine Department of Sheikh Zayed Hospital, Rahim Yar Khan.

The inclusion criteria comprised patients with T2DM who met three out of the five MetSy criteria. Patients with type 1 diabetes mellitus, age less than 20 years, newly diagnosed diabetes, secondary diabetes, pregnant women, incomplete records, and other associated comorbidities were disqualified from this study.

Data collection

After satisfying the ethical research board of Sheikh Zayed Medical College and Hospital with approval reference number 248/IRB/SZMC/SZH, a total of 160 patients fulfilling the inclusion criteria were selected. Informed consent was obtained from patients after explaining the study’s objective.

All patients were interviewed and received a printed questionnaire. The sociodemographic variables include age, gender, residence, and marital status. Clinical variables included physical activity, adherence to diet, obesity, family history, duration of diabetes, medications, concomitant risk factors, and complications. WC was measured at the umbilicus level using labeled plastic tape. BMI was assessed by dividing the weight in kilograms by the square of height in meters (kg/m^2^). Blood pressure was monitored on the right arm using a standard mercury manometer in a sitting position. In addition to fasting blood glucose tests, HDL-C and low-density lipoprotein-cholesterol (LDL-C) profiling was performed and analyzed.

The stages of diabetic retinopathy were identified using fundus examination (microaneurysm, dot and blot hemorrhages, hard exudates, cotton wool spots, macular lesions, and new vessel formation). For the assessment of diabetic neuropathy, clinical evaluations such as paresthesia, numbness, vibration, and tingling sensation were used. Similarly, the diagnosis of diabetic nephropathy was based on blood pressure assessment, kidney ultrasonography, urine tests, urinary complaints of urgency or frequency, and body swelling (hands, feet, or eyes).

Data analysis

Statistical analysis from a printed questionnaire was performed using SPSS version 22 (IBM Corp., Armonk, NY, USA). Frequencies were measured for qualitative variables. The mean values of age, gender, weight, height, BMI, WC, obesity, physical activity, dietary habits, glycemic control, diabetes duration, treatment types, FPG, blood pressure, and comorbidities (hypertension, dyslipidemia) were matched between MetSy and no MetSy groups along with the presence or absence of diabetes microvascular complications in these variable categories. Relative risk factors of associated microvascular complications were calculated with 95% confidence intervals (CIs). The p-value was calculated for diabetic patients with MetSy, and a p-value <0.05 was considered significant.

## Results

A total of 160 patients with T2DM participated in this study. In T2DM patients with MetSy, the prevalence was higher (105, 65.6%, n = 160) than in those without MetSy. The mean age was 52 years with a predominance of females 83 (51.8%, n = 160) in the 50-59-year age group (56.8%). The majority of people 156 (97.5%) were married, and 122 (76.3%) were urban dwellers. The average BMI for males was 26.70 ± 0.84 kg/m² whereas BMI for females was 29.38 ± 0.54 kg/m². Overweight and obesity actualization was 57 (35.6%) and 32 (20%), respectively.

A greater WC was found in 110 (68.7%, n = 160) patients. Females exhibited a larger WC of 93.52 ± 1.58 cm, and 48 of 83 females reported diabetes microvascular complications. Whereas in males, the frequency of WC was 89.64 ± 1.79 cm, with 36 of 77 patients reporting diabetes microvascular complications. On comparing the diabetic population with the presence or absence of MetSy, this study noted significant p-values for age, gender, high waist circumference, obesity, and BMI (Table 1).

**Table 1:** Sociodemographic and general characteristics of the studied type 2 diabetes mellitus participants (n = 160).

Variables	Categories	N (frequency, %)	Metabolic syndrome (MetSy)			Diabetes microvascular complications	
			MetSy +	MetSy -	P-value	No	Yes
Age (years)	20–39	15 (10.9)	04	11	0.03	12	3
	40–49	54 (33.7)	42	12	0.02	18	36
	50–59	91 (56.8)	59	32	0.01	46	45
Gender	Male	77 (48.1)	48	29	0.02	41	36
	Female	83 (51.9)	57	26	0.01	35	48
Residence	Urban	122 (76.2)	86	36	0.08	28	64
	Rural	38 (23.8)	19	19	0.09	18	20
Marital Status	Married	156 (97.5)	102	54	0.09	74	82
	Unmarried	4 (2.5)	02	02	0.08	2	2
Waist circumference (cm)	Male	89.64 ± 1.79 (22.5)	48	29	0.0002	41	36
	Female	93.52 ± 1.58 (30)	57	26	0.0003	35	48
Body mass index (BMI) (kg/m²)	Male	26.70 ± 0.84	48	29	0.02	41	36
	Female	29.38 ± 0.54	57	26	0.03	35	48
Obesity BMI (kg/m²)	BMI >30	32 (20)	32	00	0.03	00	32
Components of MetSy	Three or more	105 (65.6)	105	55	0.02	76	84

**Figure 1:**
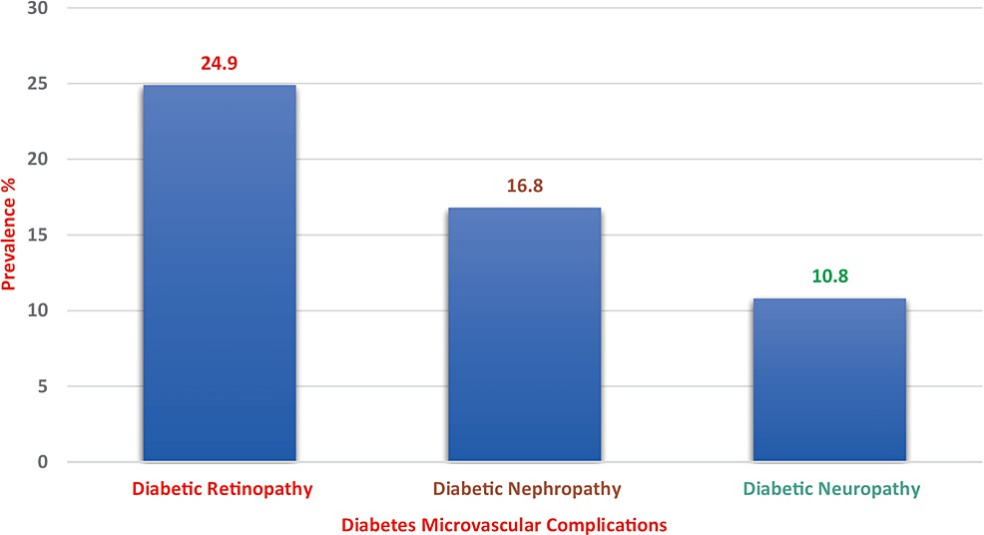
The prevalence of diabetes microvascular complications of the studied type 2 diabetes mellitus participants (n = 160).

The prevalence of microvascular complications in T2DM patients with MetSy (95% CI = 40.5%-63.6%) was 52.5% compared with 47.5% in those without MetSy. Specifically, there was a significant relationship between diabetic retinopathy prevalence of 24.9% (95% CI = 20.3%-29.6%), nephropathy prevalence of 16.8% (95% CI = 12.8%-20.7%), and neuropathy prevalence of 10.8% (95% CI = 7.4%-13.3%) in patients with T2DM (Figure 1).

According to the clinical parameters of studied T2DM participants, a family history of diabetes was present in the majority (110, 68.7%, n = 160), and 126 (78.7%) patients has a disease duration of ≥5 years. The drug of choice for 95 (59.4%) T2DM participants was oral hypoglycemic drugs (OHDs). Most of these (102, 63.7%) participants had poor physical activity as they had not exercised at all or performed less than 150 minutes/week of exercise. The majority (128, 80%) were non-obese, and 118 (73.7%) had no adherence to a balanced healthy sugar-free diet. Among these, 42 (26.3%) had adherence to diet, and 133 (83.1%) had poor glycemic control with FBS greater than 130 mg/dL. The omnipresent comorbid abnormality in T2DM patients was high blood pressure (hypertension) found in 112 (70%) participants. A significant p-value <0.05 concerning all components of MetSy (hypertension, abdominal obesity, poor glycemic control, hypertriglyceridemia, and hypo HDL) was observed (Table 2).

**Table 2:** Clinical and laboratory characteristics of the studied type 2 diabetes mellitus participants (n = 160). Good: Performed at least 150 minutes/week (three days) of moderate-intensity exercise. Poor: Had not exercised at all or performed less than 150 minutes/week of moderate-intensity exercise.

Parameters	Categories	Number (percentage %)	Metabolic syndrome (MetSy)			Diabetes microvascular complications	
			MetSy +	MetSy -	P-value	No	Yes
Family history of diabetes	Yes	110 (68.7)	84	26	0.6	63	47
	No	50 (31.3)	21	29	0.8	32	18
Diabetes duration	≥5 years	126 (78.7)	97	29	0.02	43	83
	<5 years	34 (21.3)	08	26	0.06	12	22
Medications	Oral only	95 (59.4)	51	44	0.03	31	64
	Oral + insulin	35 (21.8)	31	04	0.04	19	16
	Insulin only	30 (18.8)	23	07	0.04	9	21
Physical activity	Good	58 (36.3)	16	42	0.06	40	18
	Poor	102 (63.7)	89	13	0.003	19	83
Adherence to diet	Yes	42 (26.3)	11	31	0.05	26	16
	No	118 (73.7)	94	24	0.02	17	101
Obesity	Yes	32 (20)	30	02	0.03	4	28
	No	128 (80)	75	53	0.04	61	67
Glycemic control	Good (fasting blood sugar <130 mg/dL)	27 (16.9)	09	18	0.05	19	8
	Poor (fasting blood sugar >130 mg/dL)	133 (83.1)	96	37	0.02	25	108
Hypertension	Yes	112 (70)	92	20	0.03	9	103
	No	48 (30)	13	35	0.05	32	16
High-density lipoprotein-cholesterol	Hypo	83 (51.9)	34	49	0.004	13	70
	Hyper	77 (48.1)	71	06	0.002	46	31
Triglycerides	Hypo	74 (46.2)	26	48	0.03	43	31
	Hyper	86 (53.8)	79	07	0.0001	13	73
Components of MetSy	Three or more	105 (65.6)	105	55	0.02	76	84

MetSy was diagnosed in the study population by combining the criteria summarized in operational definitions: a combination of three criteria in 43.4%, a combination of four criteria in 36.2%, and a combination of five criteria in 20.4%.

## Discussion

Various studies have found different prevalence rates of MetSy. The IDF and AHA agree that three of the five risk factors are sufficient to establish a diagnosis of MetSy [7,8]. MetSy led to a threefold increased risk of CVDs, principally when diabetes is present in patients with MetSy [9-11]. This study aimed to estimate the prevalence, identify risk factors, and evaluate associated microvascular complications among MetSy patients with T2DM.

This study showed that MetSy prevalence in this population was 65.6% according to the new IDF 2023 definition. This statistic accorded with those reported by Saeedi et al. at 66.8% [12] and Abagre et al. at 68.8% [13]. However, the prevalence was higher than that reported in Atlanta by Ford et al. [14], with the same coordination accord at 48.9%, and by Chen et al. at 51.4% [15]. MetSy is highly prevalent in these patients, with a predominance of T2DM (78.4%), a rate comparable to that seen in the study reported by Vest et al. in 2018 at 79% [16]. Among type 1 diabetics with MetSy, 27.3% were diagnosed. The study by Vest et al. in 2018 reported a rate of 22.2% [16], and a study by Udell et al. reported a rate of 25.5% [17]. It is possible to explain these variations in MetSy prevalence by the time of the study, population, sociodemographic differences, and definitions of MetSy.

The mean age in this study was 52 years, with most participants in the 50-59-year age group (56.8%). More than half of the patients (83, 51.8%, n = 160) were females. The majority of participants (156, 97.5%) were married, and 122 (76.3%) were urban dwellers, as reported by Udell et al. [17] and Backholer et al. in the Australian population [18]. Regarding gender, the prevalence of MetSy with associated diabetic microvascular complications in this study revealed a female predominance, with a frequency of 83 (51.9%) compared to T2DM with 77 males at 48.1% (p = 0.01). These findings are similar to those reported by Fawwad et al. in Balochistan, Pakistan [19], and by Adeleye et al. in Nigeria [20]. This distinction might be related to physical inactivity, obesity, large WC, and menopause in women.

A large WC was found in 110 (68.7%, n = 160) participants. The frequency of WC in males was 89.64 ± 1.79 cm, and 36 of 77 patients reported diabetes microvascular complications. In females, the WC was 93.52 ± 1.58 cm, and 48 of 83 females reported diabetes microvascular complications. These results are in line with those reported by Dündar and Akıncı in 2022 from Turkey [21]. The average BMI for males was 26.70 ± 0.84 kg/m² whereas BMI for females was 29.38 ± 0.54 kg/m². Being overweight was noted in 57 (35.6%), and 32 (20%) participants were obese.

In this study, the majority had a family history of diabetes (110, 68.7%, n = 160), and 126 (78.7%) has a disease duration of ≥5 years. The drug of choice for 95 (59.4%) T2DM participants was OHDs. Most of these (102, 63.7%) had poor physical activity as they had not exercised at all or performed less than 150 minutes/week of exercise. The majority of them (128, 80%) were non-obese, and (118, 73.7%) had no adherence to a balanced healthy sugar-free diet. Among these, 42 (26.3%) had adherence to diet, and 133 (83.1%) had poor glycemic control with FBS greater than 130 mg/dL. The omnipresent comorbid parameter in type 2 diabetics was high blood pressure (hypertension) found in 112 (70%). Matsubayashi et al. [22] and Alshammary et al. [23] in a meta-analysis of observational studies reported that it was the most determining factor for MetSy prevalence, in contradiction to the study by Dündar and Akıncı [21] where visceral obesity was the most frequent criterion (68.3%). A significant p-value <0.05 concerning all components of MetSy (hypertension, abdominal obesity, poor glycemic control, hypertriglyceridemia, and hypo-HDL) was observed [24-25].

MetSy was diagnosed in this T2DM study population by combining the criteria summarized in operational definitions: a combination of three criteria in 43.4%, a combination of four criteria in 36.2%, and a combination of five criteria in 20.4%. These results are in line with those reported by Rossi et al. [24] and Thomas et al. [25]. The triad of diabetes, central obesity, and hypertension represented the most commonly reported correlation of MetSy in a Taiwanese study by Hsu et al. on patients with T2DM [26].

There was a 36.9% prevalence of microvascular complications in this study (95% CI = 31.5%-42.3%). Specifically, diabetic retinopathy was the most common microvascular complication, with a prevalence of 24.9% (95% CI = 20.3%-29.6%) versus 19.6% of the T2DM without MetSy (p < 0.05), which is higher than that reported by Hsu et al. in Taiwan at 37.9% [26]. Hypertension was reported in 82% of the Asian population in a study by Bhatti et al. in India [27].

Diabetic kidney disease was found in 16.8% (95% CI = 12.8%-20.7%), which is similar to that reported by de Boer et al. at 15.85 [28] in contrast to the Thomas et al. study where nephropathy was observed at a prevalence of 40.8% [25]. Diabetic neuropathy was noted at a prevalence of 10.8% (95% CI = 7.4%-13.3%) among associated MetSy patients, which is comparable to studies by Thomas et al. at 13% and Hsu et al. at 12.6% [25,26]. Ischemic heart disease was reported to be the most commonly observed macrovascular diabetes complication, with 58.3% compared to 24.7% for patients without MetSy (p < 0.05) [27], and stroke was reported at 6.6% [29,30].

There are no specific treatment modalities for MetSy in T2DM. Hence, it is crucial to effectively correct each of the variables of MetSy as soon as possible. The aim should be to modify sedentary to an active lifestyle with good adherence to a balanced diet and increased physical activity, educating on the intake of quality food (reducing excess calories), and losing excess weight, mainly abdominal girth. To reduce the impact of this disease on the microvascular system, it is important to correct these metabolic complications [27-30].

Excluding patients with type 1 diabetes mellitus, age less than 20 years, newly diagnosed diabetes, secondary diabetes, pregnant women, incomplete records, sample size, and other associated comorbidities was a limitation of this study. Nevertheless, this study was able to estimate multiple variables in a single study, which is its strong point.

## Conclusions

The prevalence of MetSy observed among T2DM patients was high (65%), with married obese females in the 50-59-year age group being more likely to be affected than males. Hypertension, poor glycemic control, high triglycerides, low HDL-C, and greater WC and BMI are additional risk factors that tend to increase the MetSy burden in T2DM. Diabetic retinopathy, nephropathy, and neuropathy are the most prevalent microvascular complications of diabetes, and immediate attention is needed to stop their detrimental effects. Longer uncontrolled diabetes, increasing age, and hypertension are independent predictors of microvascular complications. To further reduce the risks of complications that threaten healthy aging and prognosis for these patients, MetSy screening, health education, and better diabetic management are crucial.
